# Do we need a new global policy for ending preventable perinatal deaths in fragile low-income countries?

**DOI:** 10.7189/jogh.12.03020

**Published:** 2022-05-21

**Authors:** Espen Heen, Ketil Størdal, John Wachira, Ingjerd Heen, Karen M Lundeby

**Affiliations:** 1 Medical faculty, University of Oslo, Oslo, Norway; 2 Norwegian Institute of Public Health, Oslo, Norway; 3 Kenya Paediatric Association, Nairobi, Kenya; 4 Immigration health services^,^ Fredrikstad Municipality, Fredrikstad, Norway; 5 Pediatric Department, Hargeisa Group Hospital, Hargeisa, Somaliland

In 2020, an estimated 2.4 million neonates lost their lives, and an additional 2 million were stillborn. The ten countries in the world with the highest estimated neonatal mortality rate (NMR) – all above 33 per 1000 live births – have had an average annual reduction in NMR of 1.3% in the last 30 years. This decline is significantly less than the world average of 2.6% and less than all of sub-Saharan Africa at 1.7% [1,2].

The *Every Newborn Action Plan* (ENAP) is a globally-endorsed strategy for ending preventable newborn deaths that supports the Sustainable Development Goal 3.2 of an NMR of less than 12 per 1000 in every country by 2030 [3]. For this to happen, interventions are needed jointly at the community, primary, and secondary health care level, with a strong emphasis on continuity of care in reproductive-maternal-newborn-child-adolescence (RMNCA) programming.

**Figure 1:**
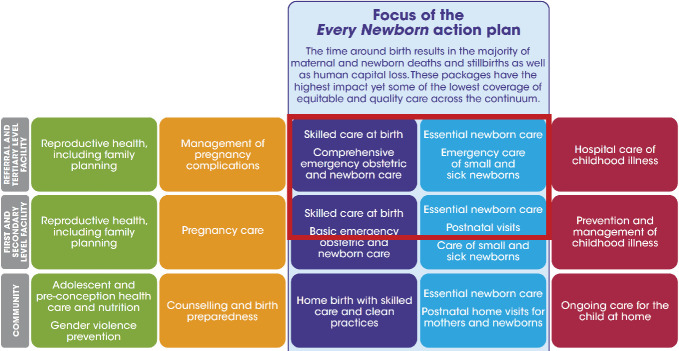
The red frame includes the components described as small and sick newborn care (SSNBC). Source: The Every Newborn Action Plan (2014), conception from The Lancet every newborn series, Mason et al. (2014).

The consortium of *Helping Newborns Survive and Helping Mothers survive* has been or is in the process of developing several well-structured, tested, and efficient interventions at all care levels in low- and lower-middle-income countries (LLMIC). However, the most complex building block has received little attention until recently: the facility-based care of small and sick newborns who are not possible to stabilize with basic care [4]. Mainly due to substandard intrapartum care in many LLMIC, there is a disproportionally large need for such neonatal treatments in the first days after birth [5]. For example, in Nairobi County in Kenya, a relatively well-functioning capital city, estimates indicate that one in five newborns needs inpatient care above what can be handled in a maternity ward [6]. The ENAP proposed that, by 2020, at least 50% of this category of newborns should receive level 2 inpatient care, in-between basic and neonatal intensive care (**Figure 1**) [3].

Recent assessments in 5 LLMIC show that only 66% of all facilities offering skilled labour care had performed some kind of resuscitation of a newborn, and a meagre 40% initiated Kangaroo Mother Care in the last three months. Newborn bag-masks were available in 59% of wards, yet only 40% had one or more employees with updated resuscitation training [7]. A faster international roll-out of the Helping Babies Breathe (HBB) concept could have improved these figures considerably. Tanzania is a shining example of how fresh stillbirths and one-day neonatal mortality can be brought down [8]. However, the uncertain HBB impact on late neonatal mortality highlights the desperate need for a broader work package [9]. One multi-country bottleneck analysis described massive challenges with all World Health Organisation (WHO) health system building blocks, particularly with medical products/technologies, financing, and health workforce of facility-based small and sick newborn care (SSNBC) [4]. Structured training leading to a neonatal nurse specialist degree is lacking in most LLMIC [10]. Combined with institutional delivery coverage in several LLMIC of less than 50%, most newborns in the world are still without access to efficient SSNBC at the beginning of 2022 [11].

**Figure:**
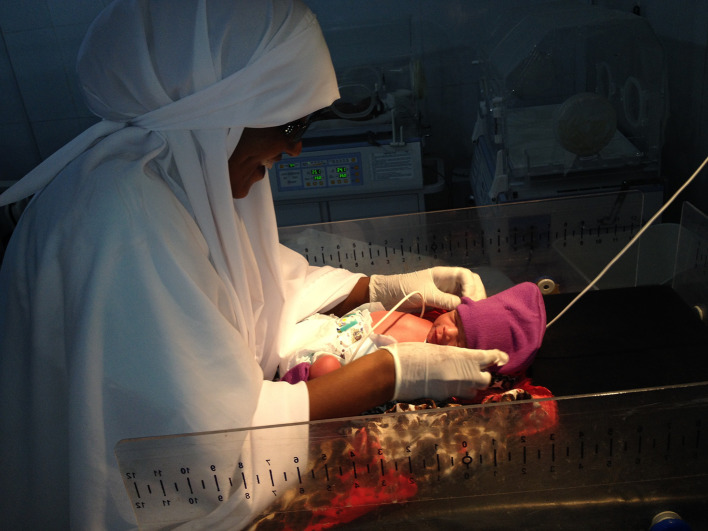
Photo: There is a large potential for improved care of small and sick newborns in fragile countries worldwide. Source: Hargeisa Group Hospital, neonatal ward. Used with permission.

Country fragility is a concept measured by the additive effects of demographic pressure, refugee-burden, tensions between groups, violence, brain drain, uneven economic development, poverty, public service, human rights, the legitimacy of the state, and the rule of law [12]. There seems to be an association between high NMR and country fragility: Of the ten countries with the highest NMR, seven were defined as *“fragile and conflict-affected situations”* by the World Bank in 2021 [2,13]. Among the 30 countries that the Fund for Peace ranks at the high end (Alert 90-120) of their 2021 fragility index, 22 have an NMR above 20 per 1000 live births [2,14]. The estimated number of newborn deaths in these 22 fragile states in 2020 was about 1.1 million, or 45% of the global number of neonatal deaths that year [2]. Except for five countries on three continents, they are all found in sub-Saharan Africa.

The most up-to-date *Every newborn progress report* (2019) provides an overview of the challenges in fragile, high-burden countries [15]. Many are still lacking the necessary policies and plans, are struggling with the ENAP reporting, and risk disappearing from the global spotlight. According to the ENAP national tracking tools, some are hardly making any progress and, according to projections based on current trends, will not reach the SDG NMR goals before the very end of this century [16].

Moving forward with SSNBC without effective top-down facilitation of policies, programs, funding, protocols, equipment, supplies, health management information systems, and training is incredibly difficult for the health managers on the ground, although not impossible, as recently shown elsewhere [17]. In our own experience, the international non-governmental community has more plans filled with technical language, more resources and tools, and higher expectations than the national and local governments possibly can absorb. Even the innovative and promising *Implementation Toolkit for Small and Sick Newborn Care,* co-chaired by the United Nations Children’s Fund (UNICEF) and Nest360, probably requires too much local competency and initiative to be a game-changer in these countries.

We believe in and want every health intervention to be owned by a country's government. Nevertheless, we also need to consider the growing gap between global aspirations and the effects of current efforts and interventions in the ten-or-so predominantly sub-Saharan, high-burden countries with low progress in NMR, fragility in several dimensions, and limited political and administrative capacity.

**Figure 2:**
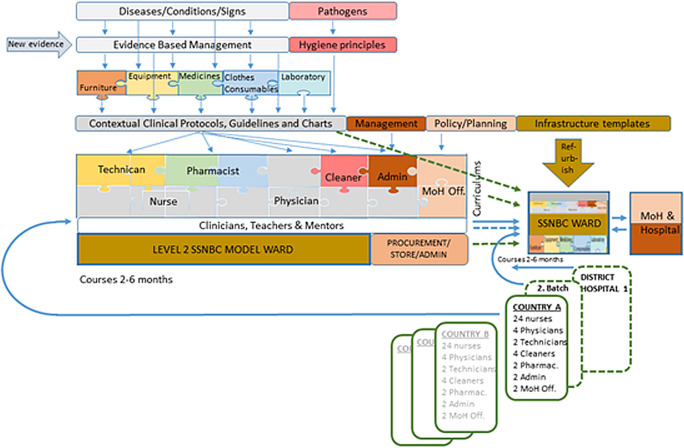
A graphical illustration of the “plug and play” concept. The coloured rectangles to the left, linked with thin blue arrows, describe the development and characterization of fully complementary soft- and hard-ware for SSNBC, the curriculums for the whole team and the first training hub with staff. The bold blue solid arrows show the flow of team training, first through the SSNBC model ward at the hub, and next, at the first SSNBC national ward in country A, where all clinical and teaching activities further will be replicated to district-level teams. Blue dashed arrows show mentoring activities and green dashed arrows show how the national SSNBC wards are equipped and organized similar to the model ward with resources from the Hub. Country B, C and D are next in line to initiate their SSNBC national programs.

What can we do differently to accelerate the complex SSNBC intervention while awaiting the long-term visions of peace, good governance, and stability in these countries? We propose a “plug and play” package of all seven health-system building blocks, rolled out from three different hubs in Western, Eastern, and Southern Africa (**Figure 2**). We already know the relevant newborn conditions and the bits and pieces of their corresponding best practice interventions. Therefore, we should be able to tailor-make sustainable furniture, identify robust medical equipment, and put together packages of neonatal clothes, consumables, and medicines – ready for order and balanced to each other and the number of deliveries per facility.

Model wards with level 2 SSNBC, linked to neonatal clinical institutions of excellence at each hub, will receive appointed physicians, nurse-midwives, pharmacists, cleaners, technicians, hospital administrators, translators, and Ministry of Health program officers from neighbouring, fragile nations. Each cadre will absorb teaching, training, and practical mentoring built on complementary curriculums. To sum up, the national teams will simultaneously learn how to build policies, administer staff, manage stocks, handle and repair equipment, translate guidelines, run a ward, and treat small, sick neonates.

Back in their home countries, the new neonatal units will be established with identical equipment, protocols, routines, charts, etc. Regular visits from regional mentors may ensure continuity, local adaptation, and integration with labour wards and obstetric care.

When robustly running, the training-of-trainers concept will help the new units duplicate the training for new staff and later spread SSNBC to new facilities in-country. The hubs could continue to manage all purchasing and logistic activity. They will also regularly update all protocols and training curricula when new evidence and equipment emerge and run virtual seminars to disperse this new knowledge to all daughter units. In this way, the national teams can focus on the clinical work and continued medical education. A “reinvention of the wheel” will be avoided. A detailed description of necessary interventions and tools when establishing a unit for SSNBC in a fragile context has just been published [18].

National governments will have to sign on to the package, build or refurbish appropriate buildings based on standard templates, and recruit, employ, and retain the neonatal staff with adequate incentives to stay long-term in the wards. Lack of political engagement may be compensated by making these agreements directly with the individual facility.

Is it doable? We cannot know, but we believe it is. What we try to envision is in many ways what India has been doing during the last 15 years: pre-designing every component in SSNBC and mass assembling a well-functioning clock, resulting in a remarkable scale-up in state after state [19]. In sub-Saharan Africa, Rwanda has seen a fast decline in NMR in parallel with implementing a broader package of interventions, including SSNBC [20]. The extra challenges with this proposal will be the multiplicity of countries and governments, the local instability, adaptation to different referral systems, and pooling of the necessary international funding. On the positive side, there are only a few structures to alter where SSNBC hardly exists, substantial cost savings in large-scale replication, and little money to waste where existing international investments in SSNBC have had little impact. The potential impact – tens of thousands of newborn lives saved before 2030.
